# Ultrasonic-Assisted Synthesis of Layered Core–Shell Ni-MOF Derivatives for Enhanced Hydrogen Sensing

**DOI:** 10.3390/nano16140858

**Published:** 2026-07-13

**Authors:** Bo Wang, Minzhe Sun, Zhenqian Cheng, Tingting Hao, Yangyang Wang, Xin Li, Hongbo Xu

**Affiliations:** 1 School of Chemistry and Chemical Engineering, Harbin Institute of Technology, Harbin 150001, China; 2 School of Physics, Harbin Institute of Technology, Harbin 150001, China; 3 The 49th Research Institute of China Electronics Technology Group Corporation, Harbin 150028, China

**Keywords:** hydrogen sensor, MOF-derived materials, core–shell structure, gas-sensing performance

## Abstract

Hydrogen sensing is of great significance for environmental monitoring and safety due to the low explosion limit and high flammability of hydrogen gas. In this work, layered and bulk Ni-MOF precursors are designed and pyrolyzed to obtain Ni-Layer-Pyrolysis and Ni-Bulk-Pyrolysis materials. Structural characterizations reveal that Ni-Layer-Pyrolysis inherits a layered morphology with a core–shell structure, higher graphitization degree, and more uniform active sites compared with its bulk counterpart. Electrochemical studies demonstrate that Ni-Layer-Pyrolysis exhibits lower charge-transfer resistance and higher carrier density, which facilitate efficient electron transport. Gas-sensing tests show that the Ni-Layer-Pyrolysis sensor achieves a low detection limit of 100 ppm, a sensitivity of 6.24 at 8000 ppm H_2_. Moreover, it displays excellent selectivity against common interfering gases and outstanding long-term stability over 40 days. These results indicate that the layered structure and core–shell architecture play a decisive role in enhancing sensitivity, selectivity, and durability. This study provides new insights into the design of MOF-derived nanostructures for high-performance hydrogen sensors with practical application potential.

## 1. Introduction

Hydrogen energy has attracted considerable attention as a key element in future sustainable energy systems due to its abundance, broad applicability, high energy density, and zero carbon emissions during utilization [1,2]. Nevertheless, the lower explosion limit of hydrogen is only 4%, and accidental leakage can easily lead to the accumulation of explosive mixtures in confined environments such as underground garages [3,4], fuel cell compartments [5], industrial pipelines [6], and manned spacecraft [7,8]. In the absence of timely detection and accurate warning, such leaks pose a significant risk of catastrophic accidents. Consequently, the development of hydrogen sensors with high sensitivity and long-term stability is of great importance in environmental monitoring and safety assurance [9,10]. Among the available sensing materials, metal oxide semiconductors such as SnO_2_ [11], NiO [12,13], and ZnO [14] have been widely explored owing to their low cost and intrinsic gas-sensing activity. However, their high operating temperatures, poor selectivity, and insufficient stability severely limit further practical applications.

In recent years, metal–organic frameworks (MOFs), with their tunable porosity and exceptionally high surface areas, have emerged as promising candidates for gas-sensing applications. For instance, Nguyen reported a Ni-doped MOF-based sensor, although the response remained unsatisfactory [15]. Similarly, Nguyen examined MOF-74 structures incorporating different metal centers (Co, Ni, Mg) for hydrogen sensing, which exhibited certain selectivity but relatively low sensitivity, with a specific surface area of 2420 m^2^/g and a response value of 58% to 50 ppm hydrogen [16]. To overcome this, increasing attention has been directed toward MOF-derived nanocomposites obtained via pyrolysis, which integrate the inherent semiconducting properties of metal oxides with the structural advantages of porous carbon frameworks. Yao et al. systematically investigated the application of MOF-derived porous materials in gas detection, specifically elucidating that MOF-derived nanostructures can effectively inherit the precursor morphology while retaining the high porosity of metal-site-rich MOFs. This facilitates high intrinsic catalytic performance, favorable redox properties, and strong synergistic effects among different components, positioning MOF-derived materials as promising candidates for next-generation high-performance gas sensors [17]. Such materials provide improved charge-transport pathways, more accessible surface areas, and enhanced catalytic activity. For example, D’Mello has developed a nitrogen-doped carbon-supported Co–ZnO hybrid material derived from MOFs. The pyrolyzed material exhibited a specific surface area of 300 m^2^/g and achieved a sensitivity of 3.7% at hydrogen concentrations below 1% [18]. Likewise, Nagy L. Torad demonstrated a three-dimensional hollow graphitic carbon network, prepared by combining graphene oxide (GO) with ZIF-8-derived porous carbon polyhedra, which exhibited superior sensitivity toward toxic aromatic compounds [19].

Despite these advances, the application of MOF-derived materials in hydrogen sensing remains at an early stage, and the influence of different precursor architectures on sensing performance has not been fully elucidated. In this study, a layered Ni–BDC complex was synthesized via an ultrasound-assisted route, followed by pyrolysis under an inert atmosphere to yield a novel Ni-Layer-Pyrolysis hybrid with a well-defined core–shell structure. The material was integrated onto a MEMS micro-hotplate platform to fabricate hydrogen sensors, enabling systematic evaluation of its gas-sensing behavior. The results revealed that the ultrasound-assisted synthesis favored the formation of layered structures with enhanced electron-transport capability, leading to improved sensitivity toward hydrogen, while the core–shell configuration contributed to superior structural robustness during prolonged operation. This work provides new insights into the design of MOF-derived nanostructures for next-generation hydrogen sensors with high sensitivity and stability.

## 2. Materials and Methods

### 2.1. Synthesis of Materials

Ni-MOF-Layer: To prepare Ni-MOF-Layer, 0.75 mmol of terephthalic acid (BDC) was ultrasonically dissolved in a mixed solvent containing 32 mL N,N-dimethylformamide (DMF), 2 mL ethanol, and 2 mL deionized water in a 100 mL Teflon vessel. Subsequently, 0.75 mmol of NiCl_2_·6H_2_O was introduced, and the solution was stirred for 5 min before the rapid addition of 0.8 mL triethylamine (TEA). After another 5 min of stirring, a colloidal suspension was formed. The sealed suspension was then subjected to ultrasonic treatment at 40 °C (40 kHz) for 8 h. The light-green precipitate was isolated by centrifugation, thoroughly washed with ethanol and deionized water (five times each), and finally freeze-dried under vacuum to yield Ni-MOF-Layer powder.

Ni-MOF-Bulk: For comparison, the above precursor solution was transferred into a 50 mL Teflon-lined autoclave and hydrothermally treated at 140 °C for 24 h. The obtained precipitate was collected by centrifugation, washed, and dried at 70 °C overnight to afford Ni-MOF-Bulk powder.

Pyrolysis Treatment: Both Ni-MOF-Layer and Ni-MOF-Bulk were subjected to pyrolysis under a N_2_ atmosphere. The samples were heated to 400 °C at a ramp rate of 5 °C/min, maintained for 2 h, and then naturally cooled to room temperature, producing Ni-Layer-Pyrolysis and Ni-Bulk-Pyrolysis, respectively.

All chemical reagents were obtained from Sinopharm Chemical Reagent Co., Ltd. (Shanghai, China). The experimental gases were supplied by Dalian Darte Gas Co., Ltd. (Dalian, China).

### 2.2. Characterization

Powder X-ray diffraction (XRD) patterns were recorded on a PANalytical Empyrean diffractometer using Cu Kα radiation (λ = 1.5418 Å) in the 2θ range of 4–60°. Scanning electron microscopy (SEM) images were obtained on a HITACHI SU8010 microscope (Tianjin, China) operated at an accelerating voltage of 5 kV with a current of 10 μA. High-resolution transmission electron microscopy (HRTEM) was performed on an FEI Tecnai G2F20 microscope (USA). Raman spectra were measured using a HORIBA XPLORA Raman spectrometer (France) equipped with a 532 nm laser, and the samples were drop-cast onto glass slides prior to testing. Electrochemical measurements were performed on a CHI660D electrochemical workstation (Shanghai, China) using a standard three-electrode configuration. The working electrode was an ITO-coated glass slide (effective area: 1.0 cm × 1.0 cm) drop-cast with the sample suspension, the counter electrode was a platinum wire, and the reference electrode was an Ag/AgCl electrode (saturated KCl). The electrolyte was 0.1 M Na_2_SO_4_ aqueous solution. Mott–Schottky measurements were conducted at room temperature with an AC amplitude of 10 mV at a fixed frequency of 1000 Hz, over a potential range from −0.8 V to +0.5 V (vs. Ag/AgCl). Electrochemical impedance spectroscopy (EIS) was performed at the open-circuit potential over a frequency range from 100 kHz to 0.01 Hz with an AC amplitude of 5 mV.

### 2.3. Gas-Sensing Test

For gas-sensing measurements, the as-prepared materials were dispersed in n-propanol (10 g/L) and drop-cast onto MEMS micro-hotplate electrodes to fabricate sensor devices. The hydrogen concentration was controlled by a dynamic gas-mixing system (HSI313). The resistance variation was continuously monitored at an optimized operating temperature of 73 °C. The sensor response was defined as:

$$S=\frac{Rg}{Ra}$$

where Ra represents the steady-state resistance of the sensor in air, Rg is the steady-state resistance in the presence of the target gas, and *S* denotes the sensor response.

Response Time and Recovery Time Measurement: The response time is defined as the time required for the baseline resistance to reach 90% of the total variation during gas adsorption when the sensor is switched from air to a hydrogen atmosphere. The recovery time is defined as the time required for the resistance to reach 90% of the total variation during gas desorption when the sensor is switched back from hydrogen to air.

### 2.4. Experimental Conditions

**Figure 1:**
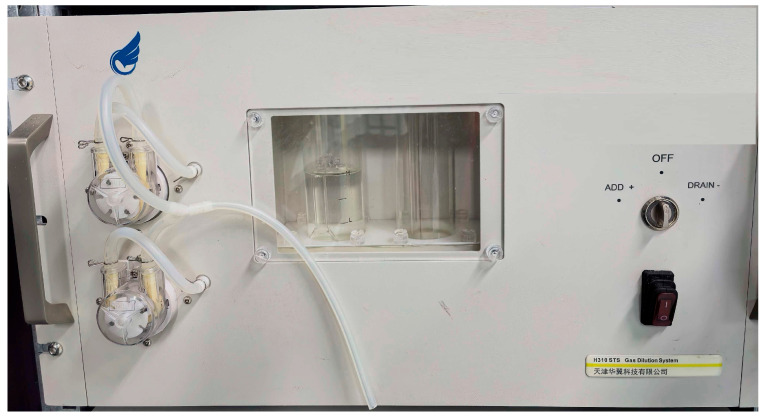
Dynamic dilution gas mixing system (HSI313).

The sensor testing was performed using an HSI313 dynamic dilution gas mixing system manufactured by Tianjin Huayi Company, shown in Figure 1. Standard gases were supplied by Dalian Date Gas Company. The equipment and gases used have been verified by standard metrology institutions to ensure data validity.

## 3. Results

### 3.1. Structural and Morphological Characterization

**Figure 2:**
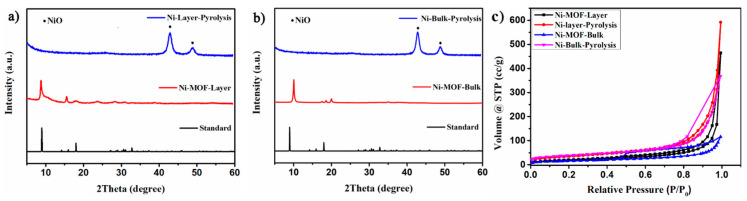
(**a**) XRD image of Ni-MOF-Layer and Ni-Layer-Pyrolysis, (**b**) XRD image of Ni-MOF-Bulk and Ni-Bulk-Pyrolysis, (**c**) N_2_ sorption studies of Ni-MOF-Layer, Ni-Layer-Pyrolysis, Ni-MOF-Bulk and Ni-Bulk-Pyrolysis.

As shown in Figure 2a,b, the XRD patterns of Ni-MOF-Layer and Ni-MOF-Bulk are consistent with the monoclinic cell structure of Ni_2_(OH)_2_BDC MOF [20]. After pyrolysis, characteristic diffraction peaks of NiO appeared at 2θ = 37.2° and 43.2°, confirming the formation of NiO phases. As shown in Figure 2c, the N_2_ adsorption-desorption studies indicate that the BET specific surface areas of Ni-Layer-Pyrolysis and Ni-Bulk-Pyrolysis are 388 and 180 m^2^·g^−1^, respectively. Although the specific surface area of the materials decreased after pyrolysis, both materials still exhibit higher surface areas than conventional metal oxide nanoparticles.

**Figure 3:**
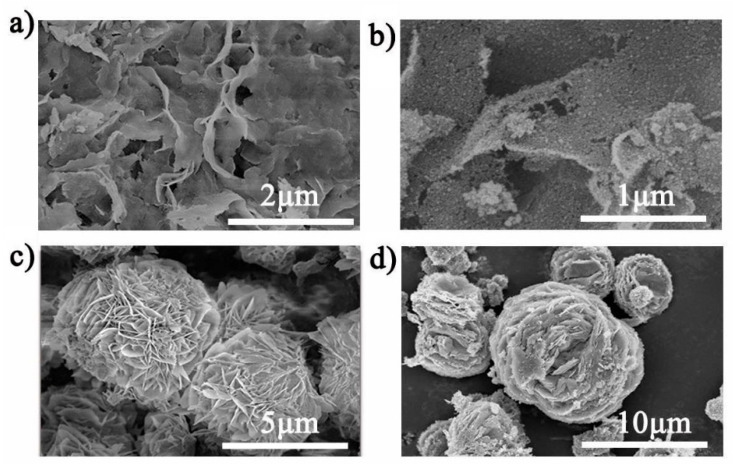
SEM image of (**a**) Ni-MOF-Layer, (**b**) Ni-Layer-Pyrolysis, (**c**) Ni-MOF-Bulk, (**d**) Ni-Bulk Pyrolysis.

The SEM images (Figure 3) indicate that Ni-Layer-Pyrolysis successfully inherited the layered morphology of its precursor, consisting of stacked carbon nanospheres, whereas Ni-Bulk-Pyrolysis exhibited a bulk-like aggregated structure.

**Figure 4:**
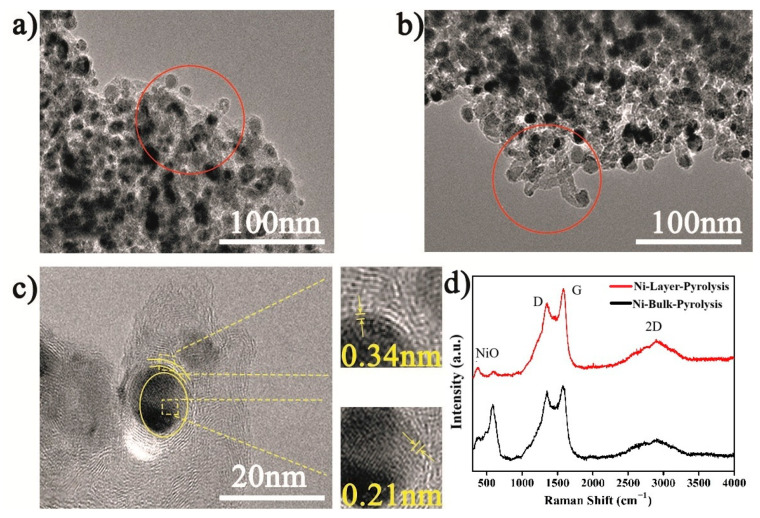
(**a**) TEM image of Ni-Layer-Pyrolysis; (**b**) TEM image of Ni-Bulk-Pyrolysis; (**c**) HRTEM image of Ni-Layer-Pyrolysis; (**d**) Raman curve of Ni-Layer-Pyrolysis and Ni-Bulk-Pyrolysis.

Further insights are provided by TEM observations (Figure 4). In Figure 4a, Ni-Layer-Pyrolysis is revealed to be composed of nanospherical particles arranged into a sheet-like structure with a core–shell configuration. In contrast, Figure 4b shows that Ni-Bulk-Pyrolysis predominantly consists of blocky assemblies formed by carbon nanotubes with Ni nanoparticles serving as nucleation centers. Importantly, as shown in Figure 4c, the NiO nanoparticles in Ni-Layer-Pyrolysis exhibit an average diameter of ~9 nm and are encapsulated within graphitic carbon shells. The observed lattice spacings of 0.24 nm and 0.34 nm correspond to the (111) plane of NiO and the (002) plane of graphitic carbon, respectively. To further confirm the core–shell architecture, high-resolution TEM images were acquired from multiple representative regions of both Ni-Layer-Pyrolysis and Ni-Bulk-Pyrolysis samples (Figures S1 and S2), which clearly reveal the layered morphology and its structural distinction from the bulk-derived counterpart. In addition, EDS line-scan analysis was conducted across individual particles (Figure S3). The results show distinct differences in Ni and O distributions between the edge and interior regions, which further support the core–shell feature.

In Figure 4d, the Raman analysis reveals that the IG/ID ratio of Ni-Layer-Pyrolysis (0.98) is higher than that of Ni-Bulk-Pyrolysis (0.89), indicating a higher degree of graphitization within the carbon matrix derived from the layered MOF precursor. Such highly graphitized carbon shells play multiple positive roles in enhancing the gas-sensing performance. First, they construct a highly conductive network surrounding the NiO nanoparticles, which serves as express pathways for electron transport, effectively reducing interparticle contact resistance and thereby facilitating rapid charge-carrier migration within the sensing layer. Second, the abundant oxygen-containing functional groups and structural defect sites on the carbon shell surface act as preferential adsorption sites for hydrogen molecules, which is favorable for improving the response sensitivity of the sensing material. Furthermore, the graphitic carbon shells effectively suppress the agglomeration and grain growth of NiO nanoparticles during prolonged operation, thus ensuring structural stability and long-term durability. Previous studies have demonstrated that metal oxide/carbon composite interfaces can significantly promote the adsorption behavior of hydrogen molecules and the subsequent charge-transfer processes [21]. XPS analysis was further conducted to investigate the surface chemical composition of the two materials. The XPS survey spectra (Figure S4) reveal that the Ni-Layer-Pyrolysis sample exhibits a higher oxygen concentration compared with Ni-Bulk-Pyrolysis, which is closely correlated with its enhanced sensing performance. In summary, the synergistic effect between the highly graphitized carbon shells and the NiO cores in Ni-Layer-Pyrolysis provides more efficient charge-transport pathways and a greater number of active sites for the gas-sensing reaction, which is closely correlated with its superior hydrogen-sensing performance. This is consistent with recent findings that MOF-derived metal oxide/carbon composites exhibit significantly enhanced gas-sensing performance, where different interfacial structures play a crucial role in modulating the sensing behavior [22,23].

### 3.2. Electrochemical Properties

To further elucidate the influence of structural differences on electron-transfer capability, the electrochemical impedance spectra (EIS) and Mott–Schottky analyses of the two catalysts were investigated. EIS was employed to characterize the charge-transfer resistance.

**Figure 5:**
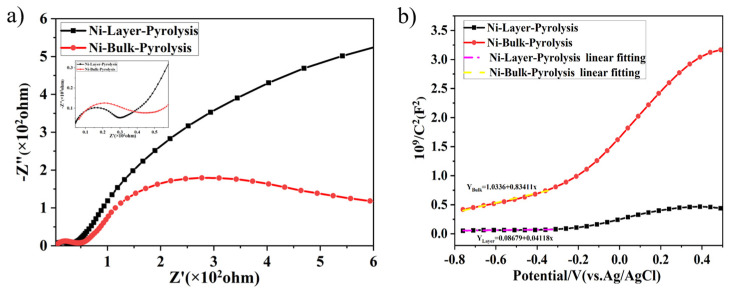
Electrochemical performance curve (**a**) Electrochemical impedance spectroscopy; (**b**) Mott–Schottky.

To further elucidate the influence of structural differences on electron transport capability, electrochemical impedance spectroscopy (EIS) and Mott–Schottky analyses were conducted on both materials. EIS was employed to characterize the charge-transfer resistance at the electrode–electrolyte interface. As shown in the enlarged high-frequency region of Figure 5a, the semicircular diameter of the Ni-Layer-Pyrolysis sample is markedly smaller than that of the Ni-Bulk-Pyrolysis counterpart, indicating a substantially lower charge-transfer resistance for the layered-derived material. This observation suggests that the layered architecture endows the material with enhanced electrical conductivity, which facilitates rapid carrier migration during the sensing process.

Furthermore, Mott–Schottky analysis was performed to evaluate the carrier concentrations of the two samples, as shown in Figure 5b. By applying linear fitting to the linear segments of the Mott–Schottky plots, the slopes were determined to be 0.0412 for Ni-Layer-Pyrolysis and 0.83411 for Ni-Bulk-Pyrolysis. According to the Mott–Schottky equation for p-type semiconductors, the carrier (acceptor) concentration is inversely proportional to the slope of the linear region. Consequently, the significantly smaller slope of Ni-Layer-Pyrolysis corresponds to a higher carrier density [24]. This result further demonstrates that the layered structure effectively promotes carrier concentration, thereby providing a greater number of active sites and more efficient charge-transport pathways for gas-sensing reactions.

In summary, the EIS results reveal the advantage of the layered structure in reducing charge-transfer resistance [25], while the Mott–Schottky analysis elucidates the underlying mechanism from the perspective of carrier density. Collectively, these findings indicate that the unique structural features of Ni-Layer-Pyrolysis synergistically enhance the charge-transport kinetics, which is closely correlated with its superior gas-sensing performance.

### 3.3. Gas-Sensing Performance

**Figure 6:**
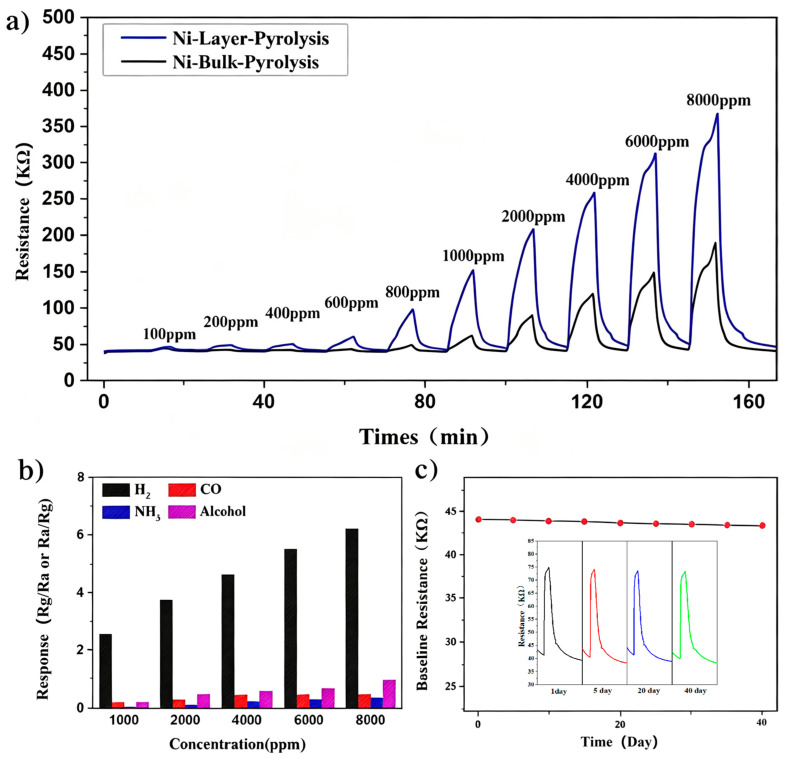
(**a**) Dynamic response-recovery curves under 100 ppm to 8000 ppm H_2_, (**b**) Selectivity measurement toward interfering gases, (**c**) Long-term stability upon exposure to 800 ppm H_2_.

Before discussing the results, we first clarify the rationale for the pyrolysis temperature selection. As shown in Figure S5c, the sensor prepared at 400 °C exhibits the highest sensitivity along with a relatively low baseline resistance. Accordingly, 400 °C was selected as the optimal pyrolysis temperature for all subsequent performance evaluations in this study. As indicated by preliminary testing, the sensor sensitivity initially increases with rising temperature and then reaches a plateau. Considering the influence of both sensitivity and baseline resistance, the sensor heating temperature was ultimately controlled at 73 °C for subsequent performance tests. Figure 6a shows the real-time resistance responses of Ni-Layer-Pyrolysis and Ni-Bulk-Pyrolysis sensors to H_2_ concentrations ranging from 100 to 8000 ppm. Both samples exhibited increased resistance with rising hydrogen concentration. However, Ni-Bulk-Pyrolysis displayed negligible variation below 800 ppm, whereas Ni-Layer-Pyrolysis exhibited a distinct and continuous response across the full range. This indicates that Ni-Layer-Pyrolysis possesses superior low-concentration detection capability and overall higher sensitivity. Benefiting from enhanced electron mobility, Ni-Layer-Pyrolysis achieved a detection limit as low as 100 ppm and a sensitivity of 6.24 at 8000 ppm H_2_. In addition, consecutive response-recovery cycle tests were also conducted on the Ni-Layer-Pyrolysis sensor at a hydrogen concentration of 1000 ppm (Figure S6), revealing a response time of 62 s and a recovery time of 31 s.

Selectivity tests were conducted by exposing the sensor to interfering gases, including CO, NH_3_, and ethanol. As shown in Figure 6b, Ni-Layer-Pyrolysis exhibited negligible response to NH_3_ (1000 ppm), and the responses to ethanol and CO (1000 ppm) were only 0.2, much lower than the response of 2.56 observed for 1000 ppm H_2_. At higher concentrations, the CO response approached saturation (≈0.5), while the ethanol response increased slightly. Notably, even at 8000 ppm, the response to H_2_ remained more than six times greater than that to ethanol. These results confirm that Ni-Layer-Pyrolysis exhibits excellent selectivity toward hydrogen, which can be attributed to its relatively low carrier density that favors selective interaction with reducing gases.

Long-term stability was further evaluated by monitoring the sensor’s response to 800 ppm H_2_ over a 40-day period (Figure 6c). The baseline resistance fluctuation remained within 1%, and the dynamic responses were well maintained throughout the aging test, highlighting the outstanding durability of Ni-Layer-Pyrolysis.

Taken together, Ni-Layer-Pyrolysis exhibits remarkable hydrogen-sensing characteristics, including high sensitivity, rapid response/recovery, low detection limit, excellent selectivity, and outstanding long-term stability. These competitive features underscore its strong potential for practical hydrogen detection applications. To further evaluate the practical applicability of the sensors, sensor-to-sensor reproducibility and multi-cycle repeatability were systematically investigated. Gas-sensing measurements on multiple independently fabricated sensor devices (Figure S5a) demonstrate that different devices exhibit good consistency in their responses to the same hydrogen concentration. Moreover, four consecutive sensing cycles at 1000 ppm, 4000 ppm, and 8000 ppm H_2_ were conducted, and the relative standard deviation (RSD) of the response values at each concentration was calculated. The results show that the maximum deviation is only 0.018 (Figure S5b), indicating that the sensor exhibits good response repeatability and stability over multiple cycles.

It can be observed from the data in the Table 1 that the Ni-Layer-Pyrolysis material prepared in this work exhibits excellent sensing performance toward hydrogen. Its response to 1000 ppm hydrogen is more than twice that of conventional nickel oxide nanoparticles, while the operating temperature is reduced from the traditional 200–300 °C to 73 °C.

## 4. Discussion

In this study, layered and bulk Ni-MOF precursors were rationally designed and pyrolyzed to obtain a novel Ni-Layer-Pyrolysis composite with a core–shell architecture. Comprehensive characterizations demonstrated that Ni-Layer-Pyrolysis possesses a higher degree of graphitization, superior electron-transport capability, and a more uniform distribution of active sites compared with its bulk counterpart. These structural advantages enabled the sensor to achieve highly sensitive, selective, and stable detection of hydrogen.

Overall, this work provides new insights into the structural design of MOF-derived materials for gas-sensing applications and establishes a solid experimental basis for the development of next-generation hydrogen sensors. The findings not only enrich the understanding of precursor–structure–performance relationships but also highlight the promising potential of MOF-derived nanostructures in practical sensing technologies.

## Figures and Tables

**Table 1:** Comparison of material properties with those reported in the literature.

Nanostructures	Concentration (ppm)	Temperature (°C)	Response	Response Time (s)	Recovery Time (s)	Ref
NiO nanowires	1000	200	1.03	659	900	[26]
NiO	500	300	1.2	20–30	20–30	[27]
NiO Nanostructure	1000	400	1.03	56	21	[28]
Ni-MOF-I	50	200	1.3	—	—	[18]
Ni-MOF-II	50	200	2.8	—	—	[29]
WO3/MWNTsc/Pd	-	200	8	—	—	[29]
Pd/Graphene q·dsd/WO_3_	3600	120	5	~108	~331	[30]
WO_3_	1300	300	1.32	340	—	[31]
Pd-coated ZnO nanorods	500	RT	4.2	—	—	[32]
ZnO-Pd nanoparticle	4000	RT	10	—	—	[33]
Co–ZnO–N/C	10,000	RT	3.7	17–26	17–26	[16]
NiO/NiWO_4_/WO_3_	50	300	0.1	—	—	[34]
Ni-Layer-Pyrolysis	1000	73	2.56	62	31	This work

## Data Availability

The data presented in this study are available on request from the corresponding author.

## Supplementary Materials

The following supporting information can be downloaded at: https://www.mdpi.com/article/10.3390/nano16140858/s1, Figure S1. TEM images and corresponding particle size distributions of Ni-Layer-Pyrolysis. Figure S2. TEM images and corresponding particle size distributions of Ni-Bulk-Pyrolysis. Figure S3. EDS elemental mapping analysis: (a) C element distribution in Ni-Layer-Pyrolysis; (b) Ni element distribution in Ni-Layer-Pyrolysis; (c) C element distribution in Ni-Bulk-Pyrolysis; (d) Ni element distribution in Ni-Bulk-Pyrolysis. Figure S4. XPS survey spectra of (a) Ni-Bulk-Pyrolysis and (b) Ni-Layer-Pyrolysis. Figure S5. Gas-sensing performance of Ni-Layer-Pyrolysis: (a) reproducibility tests; (b) repeatability error of sensitivity at different H_2_ concentrations; (c) effect of pyrolysis temperature on baseline resistance and sensitivity. Figure S6. Response and recovery time measurements of Ni-Layer-Pyrolysis: (a,b) two consecutive response curves; (c,d) two consecutive recovery curves.
