# Supplementary material for: Ultrasonic-Assisted Synthesis of Layered Core–Shell Ni-MOF Derivatives for Enhanced Hydrogen Sensing
Source: Nanomaterials (Basel). 2026 Jul 13;16(14):858. doi: 10.3390/nano16140858 (PMC13415105; doi:10.3390/nano16140858)
Supplement: Supplementary file 1 [file nanomaterials-16-00858-s001.zip › nanomaterials-4381724-supplementary.pdf]

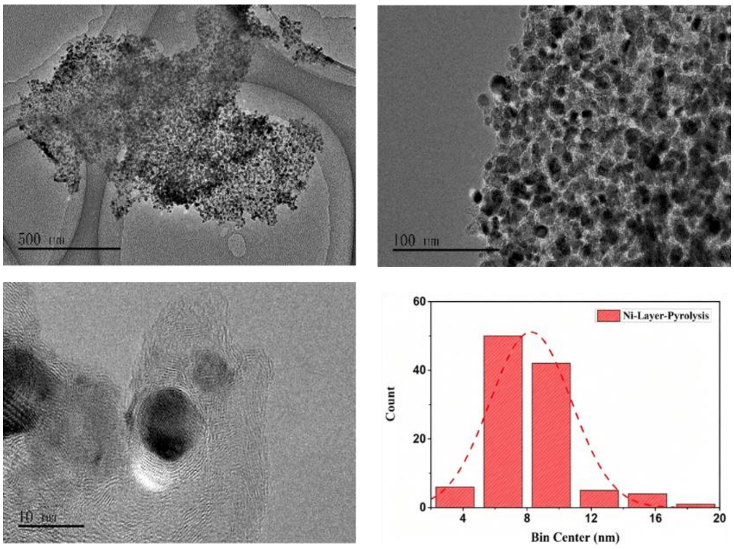


Figure S1. TEM images and corresponding particle size distributions of Ni-Layer-Pyrolysis.

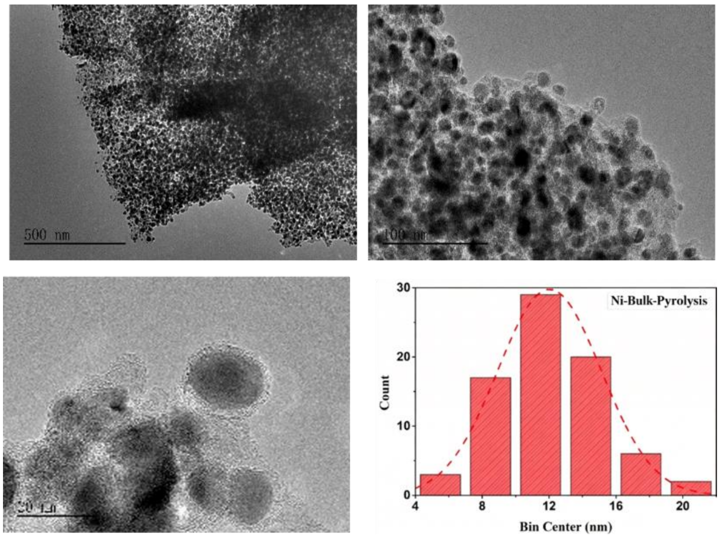


Figure S2. TEM images and corresponding particle size distributions of Ni-Bulk-Pyrolysis.

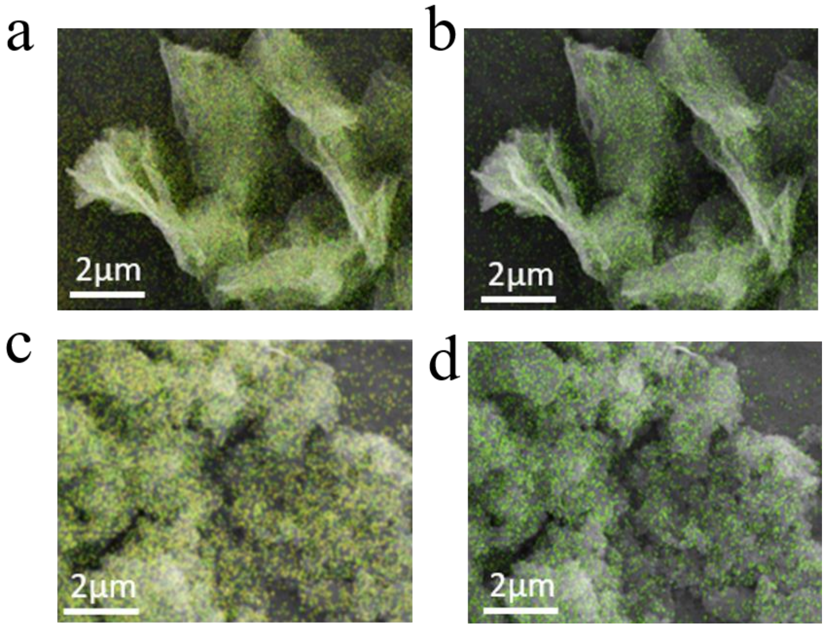

Figure S3. EDS elemental mapping analysis: (a) C element distribution in Ni-Layer-Pyrolysis; (b) Ni element distribution in Ni-Layer-Pyrolysis; (c) C element distribution in Ni-Bulk-Pyrolysis; (d) Ni element distribution in Ni-Bulk-Pyrolysis.

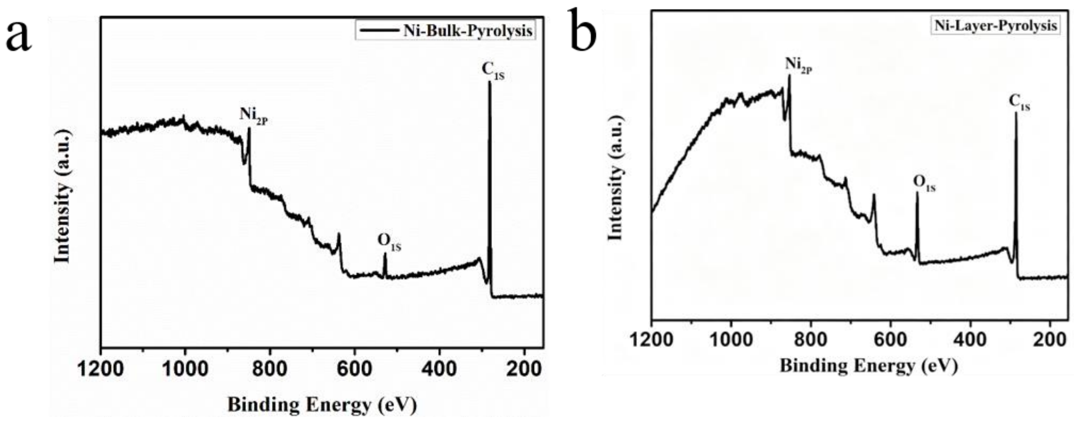

Figure S4. XPS survey spectra of (a) Ni-Bulk-Pyrolysis and (b) Ni-Layer-Pyrolysis.

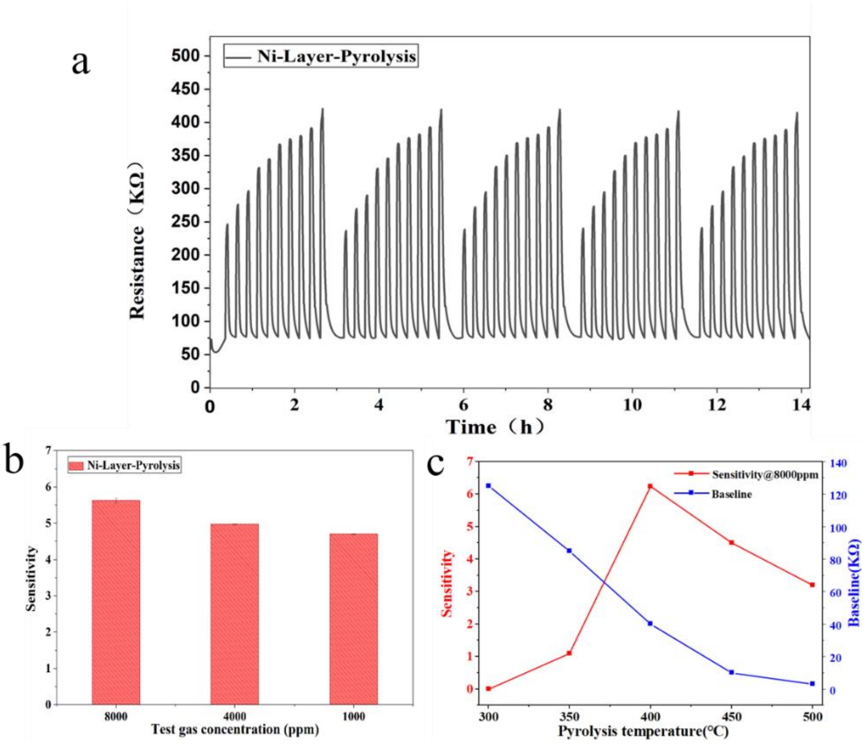


Figure S5. Gas-sensing performance of Ni-Layer-Pyrolysis: (a) reproducibility tests; (b) repeatability error of sensitivity at different $H_2$ concentrations; (c) effect of pyrolysis temperature on baseline resistance and sensitivity.

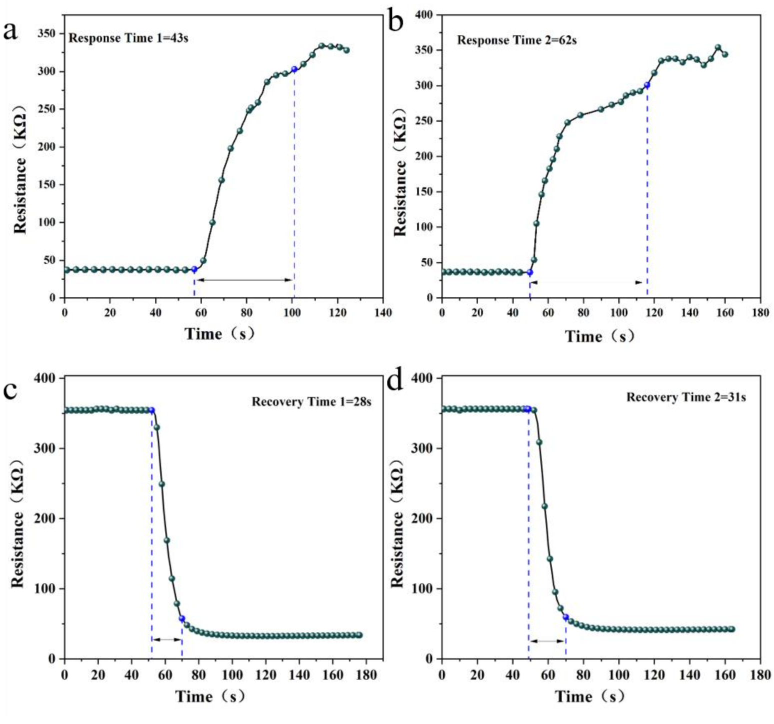


Figure S6. Response and recovery time measurements of Ni-Layer-Pyrolysis: (a,b) two consecutive response curves; (c,d) two consecutive recovery curves.
